# Hippocampal neurogenesis regulates recovery of defensive responses by recruiting threat- and extinction-signalling brain networks

**DOI:** 10.1038/s41598-019-39136-y

**Published:** 2019-02-27

**Authors:** Alonso Martínez-Canabal, Grecia López-Oropeza, Abril Gaona-Gamboa, Paola Ballesteros-Zebadua, Olinca Galvan de la Cruz, Sergio Moreno-Jimenez, Francisco Sotres-Bayon

**Affiliations:** 1 Instituto de Fisiología Celular - Neurociencias, Universidad Nacional Autónoma de México, 04510 Ciudad de México, Mexico; 2 Instituto Nacional de Neurología y Neurocirugía – Radioneurocirugía, Ciudad de México, Mexico

## Abstract

Safe exposure to a context that was previously associated with threat leads to extinction of defensive responses. Such contextual fear extinction involves the formation of a new memory that inhibits a previously acquired contextual fear memory. However, fear-related responses often return with the simple passage of time (spontaneous fear recovery). Given that contextual fear and extinction memories are hippocampus-dependent and hippocampal neurogenesis has been reported to modify preexisting memories, we hypothesized that neurogenesis-mediated modification of preexisting extinction memory would modify spontaneous fear recovery. To test this, rats underwent contextual fear conditioning followed by extinction. Subsequently, we exposed rats to an enriched environment or focal X-irradiation to enhance or ablate hippocampal neurogenesis, respectively. Over a month later, rats were tested to evaluate spontaneous fear recovery. We found that enhancing neurogenesis after, but not before, extinction prevented fear recovery. In contrast, neurogenesis ablation after, but not before, extinction promoted fear recovery. Using the neuronal activity marker c-Fos, we identified brain regions recruited in these opposing neurogenesis-mediated changes during fear recovery. Together, our findings indicate that neurogenesis manipulation after extinction learning modifies fear recovery by recruiting brain network activity that mediates the expression of preexisting contextual fear and extinction memories.

## Introduction

To survive in a dynamic environment, animals must be able to distinguish between a dangerous and a safe place. When a place is associated with an aversive consequence (e.g. a foot-shock) the brain forms a threat memory to that context (contextual fear memory), representing a dangerous place. After repeated re-exposure to the same context but without the aversive consequence (i.e. without foot-shock), the brain forms an extinction memory to that context, representing a safe place^1,2^. Later, exposure to the context evokes the extinction memory rather than fear memory, as indicated by low defensive responses to the threat. However, with the simple passage of time after extinction learning, expression of the fear memory returns, as indicated by high defensive responses to the threat^3–5^. This time-dependent phenomenon, known as spontaneous recovery of fear^6,7^, is accentuated in patients with fear-related disorders^8^. Despite the importance of spontaneous fear recovery to study how animals distinguish between safe and dangerous contexts over time and how dysregulation of this phenomenon may relate to psychopathology, its underlying brain mechanisms are not clear.

Neural activity in the hippocampus is crucial for encoding contextual memories in rodents as in humans^1^. Recent reports show that the addition of new neurons (neurogenesis) to the adult dentate gyrus of the hippocampus can modify preexisting hippocampal-dependent memories^9–12^. Contextual memory that is hippocampus-dependent, and that is crucial for survival, involves encoding the association of a place with its biological significance, as in fear and extinction memories^1,3,13^. It has been suggested that hippocampal neurogenesis modulates the expression of fear-related memories. Yet the accumulated evidence that supports this notion is conflicting. On one hand, evidence shows that facilitation of neurogenesis can increase fear context-specificity^14^, decrease context-mediated fear-related responses^9,10^ or have no effect^15^. On the other hand, there is evidence that blockade of neurogenesis can increase fear-related responses in infants^10^, decrease fear-related responses in adults^16^ or have no effect^17–19^. It is not clear whether the conflicting evidence in these different studies is due to neurogenesis manipulations being performed at different time points with respect to the formation of contextual fear and extinction memories. Thus, the time at which new neurons are added or not to the hippocampus circuitry may be crucial to understand its contribution to regulation of fear-related behaviours.

Spontaneous recovery of fear may be a particularly relevant phenomenon to study fear-related behaviours and contextual memory discrimination^2,5,20^. Testing memory retrieval during spontaneous recovery may allow to evaluate a time point when fear and extinction memories compete for the control of fear-related behaviours. Thereby this time point denotes a moment where the individual is required to distinguish between a dangerous and a safe place that may share some features^2,21^. Notably, this cognitive function (pattern separation), important for survival and mental health, has been associated with the addition of new neurons to the hippocampal circuits^22,23^. We hypothesized that a change in the hippocampal circuitry mediated by the addition or ablation of new-born neurons would modify the circuits that support fear expression during spontaneous recovery. Indeed, we found that increasing neurogenesis using environmental enrichment (EE) after, but not before or in absence of, fear extinction learning prevents spontaneous fear recovery. Consistently, we found that ablating hippocampal neurogenesis using focal X-irradiation^15^ after, but not before or in the absence of, fear extinction learning promotes spontaneous fear recovery. Notably, using c-Fos expression as a neuronal activity marker, we identified that underlying brain activity involved in neurogenesis-mediated control of fear recovery levels involves opposing recruitment in the prefrontal-amygdala-habenula network.

## Results

### Environmental enrichment after extinction increases neurogenesis and prevents fear recovery

To test the hypothesis that adding more adult-born hippocampal neurons prevents fear recovery, we compared spontaneous fear recovery levels (freezing and bar presses per minute) before and after a month of either living in EE or in a standard home cage (non-EE). On day 1, rats were conditioned to fear a context (chamber) by pairing it with an aversive consequence (mild foot-shocks). The next day (day 2), rats extinguished their fear to the conditioned context by not receiving any aversive consequence. One day later (day 3), rats were tested for memory retrieval to the context. We used this first retrieval test to match groups (experimental and control) based on similar fear response levels. This matching allows for a fair comparison of fear response levels before and after living in an EE or non-EE. After matching, fear-related behaviours (freezing and presses per minute) were similar between groups during conditioning (day 1; freezing: *F*_(1,19)_ = 1.80, *p* = 0.31; bar-pressing: *F*_(1,19)_ = 0.31, *p* = 0.58), extinction (day 2; freezing: *F*_(1,19)_ = 1.15, *p* = 0.29; bar-pressing: *F*_(1,19)_ = 1.24, *p* = 0.30) and retrieval test (test 1 on day 3; freezing: *t*_(19)_ = 0.52, *p* = 0.60; bar-pressing: *t*_(19)_ = 0.4, *p* = 0.96). Over a month later rats were evaluated for a second retrieval test (day 38).

**Figure 1:**
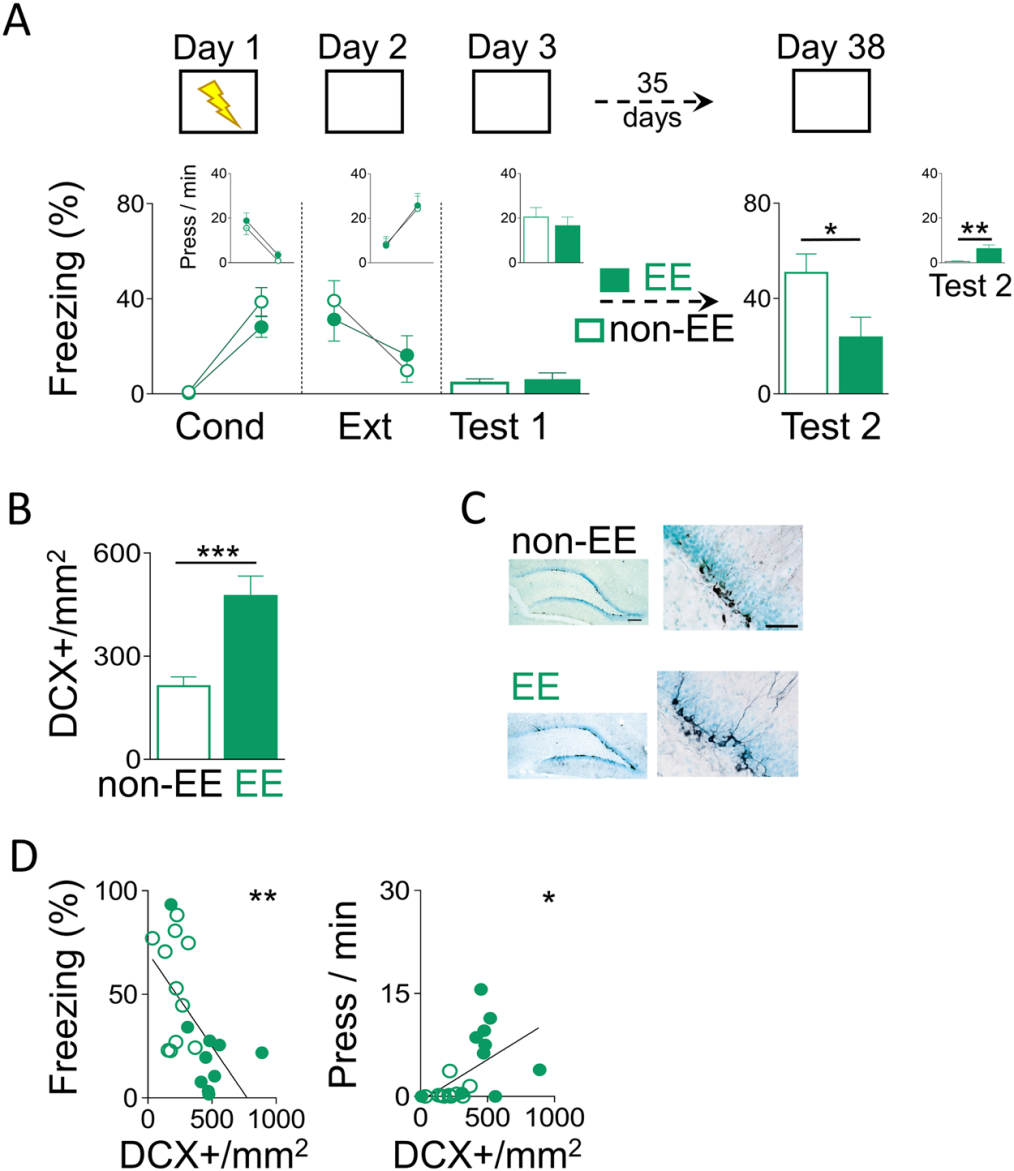
EE-mediated neurogenesis increase after extinction prevented spontaneous fear recovery. (**A**) Top, Rats were trained to associate a context with foot-shocks (day 1; conditioning). One day later, rats were trained to associate the same context with the absence of foot-shocks (day 2; extinction). The next day, rats were placed in the same context without foot-shocks (day 3; test 1). Then, rats were housed for over a month (35 days) either in an EE or a non-EE. Finally, rats were placed again in the same context alone to test for fear recovery (day 38; test 2). Bottom, EE after extinction training prevented spontaneous fear recovery, as indicated by low freezing and high bar presses per minute, when comparing EE with non-EE (EE: n = 10; non-EE: n = 11). Insets: bar-pressing behavior (press/min) across the different phases of the behavioral protocol. Data are shown for first block (early) and last block (late) of conditioning and extinction (5-minute blocks), and as a single block in test sessions (10-minute block). (**B**) Rats that lived in an EE for over a month showed more number of immature cells (DCX+) per area (mm^2^) in the dentate gyrus of the hippocampus than rats that lived in a non-EE. (**C**) Representative immunohistochemically stained immature cells (DCX+; black) in the dentate gyrus of the hippocampus after EE or non-EE. Low magnification scale bar = 200 µm (left) and high magnification scale bar = 50 µm (right). (**D**) Negative correlation between number of DCX+/mm^2^ and percent freezing response in Test 2 (R = −0.59; *p* < 0.01). Positive correlation between number of DCX+/mm^2^ and presses per min in Test 2 (R = 0.52; *p* < 0.05). *p < 0.05, **p < 0.01, ***p < 0.001. Error bars indicate SEM. EE: enhanced environment; Cond: conditioning; Ext: extinction; DCX+: doublecortin positive cells.

Rats either lived in an EE or in a non-EE for more than a month (35 days), the time that has been shown to be sufficient for adult-born new neurons to incorporate into hippocampal circuits^24^. On day 38, to evaluate spontaneous recovery of fear, rats were tested in the same context for a second retrieval test (test 2). We found, that rats that lived in an EE showed significantly lower levels of fear-related responses during test 2 as compared to rats that lived in a non-EE (freezing: EE: 23.72%; non-EE: 50.78%; *t*_(19)_ = 2.33, *p* = 0.03; bar-pressing: EE: 6.34 press/min; non-EE: 0.55 press/min; *t*_(19)_ = 3.56, *p* = 0.002), suggesting prevention of fear recovery (Fig. 1A). Moreover, rats that lived in an EE showed a robust increase in hippocampal neurogenesis, indicated by significantly higher numbers of doublecortin (DCX) positive cell density (+/mm^2^), compared to rats that lived in a non-EE (Fig. 1B,C; EE: 475.47 DCX+/mm^2^; non-EE: 212.70 DCX+/mm^2^; *t*_(19)_ = 3.56, *p* = 0.002). Importantly, this effect was associated with specific local increase of hippocampal neurogenesis by EE as indicated by lack of effect on neurogenesis in the rostral migratory stream (EE: 0.53 ± 0.09 optical density units (OD), non-EE: 0.58 ± 0.03 OD; *t*_(14)_ = 0.33, *p* = 0.74). Further, we found that during test 2, fear response levels and levels of neurogenesis were related, as indicated by DCX+ neurons correlation with freezing (R = −0.59, *p* = 0.0018) and bar-pressing (R = 0.52, *p* = 0.014) (Fig. 1D). Together, these findings indicate that EE-mediated increase of hippocampal neurogenesis after extinction prevents fear recovery.

### Hippocampal irradiation after extinction ablates neurogenesis and promotes fear recovery

Even though we showed that EE-mediated neurogenesis after extinction is sufficient to prevent fear recovery, it is possible that neurogenesis is not necessary to modulate fear recovery. To test this possibility, we ablated hippocampal neurogenesis by focal X-irradiation. To evaluate the effect of neurogenesis ablation on fear recovery, we compared spontaneous fear recovery levels (freezing and bar presses per minute) before and after three irradiation (IRR) or sham irradiation (non-IRR) sessions that occurred during a 35-day period to be comparable to the EE experiment above. Thus, on day 1, rats were conditioned to fear a context (chamber) by pairing it with an aversive consequence (mild foot-shocks). The next day (day 2), rats extinguished their fear to the conditioned context by not receiving any aversive consequence. One day later (day 3), rats were tested for memory retrieval to the context. We used this first test to match groups (experimental and control) based on similar fear response levels. This matching allows for a fair comparison of fear response levels before and after IRR or non-IRR. After matching, fear-related behaviour (freezing and presses per minute) was similar between groups during conditioning (day 1; freezing: *F*_(1,19)_ = 0.47, *p* = 0.79; bar-pressing: *F*_(1,19)_ = 0.38, *p* = 0.95), extinction (day 2; freezing: *F*_(1,19)_ = 0.13, *p* = 0.73; bar-pressing: *F*_(1,19)_ = 0.20, *p* = 0.65) and retrieval test (test 1 on day 3; freezing: *t*_(19)_ = 0.28, *p* = 0.77; bar-pressing: *t*_(19)_ = 1.27, *p* = 0.21). Then rats received either IRR or non-IRR.

**Figure 2:**
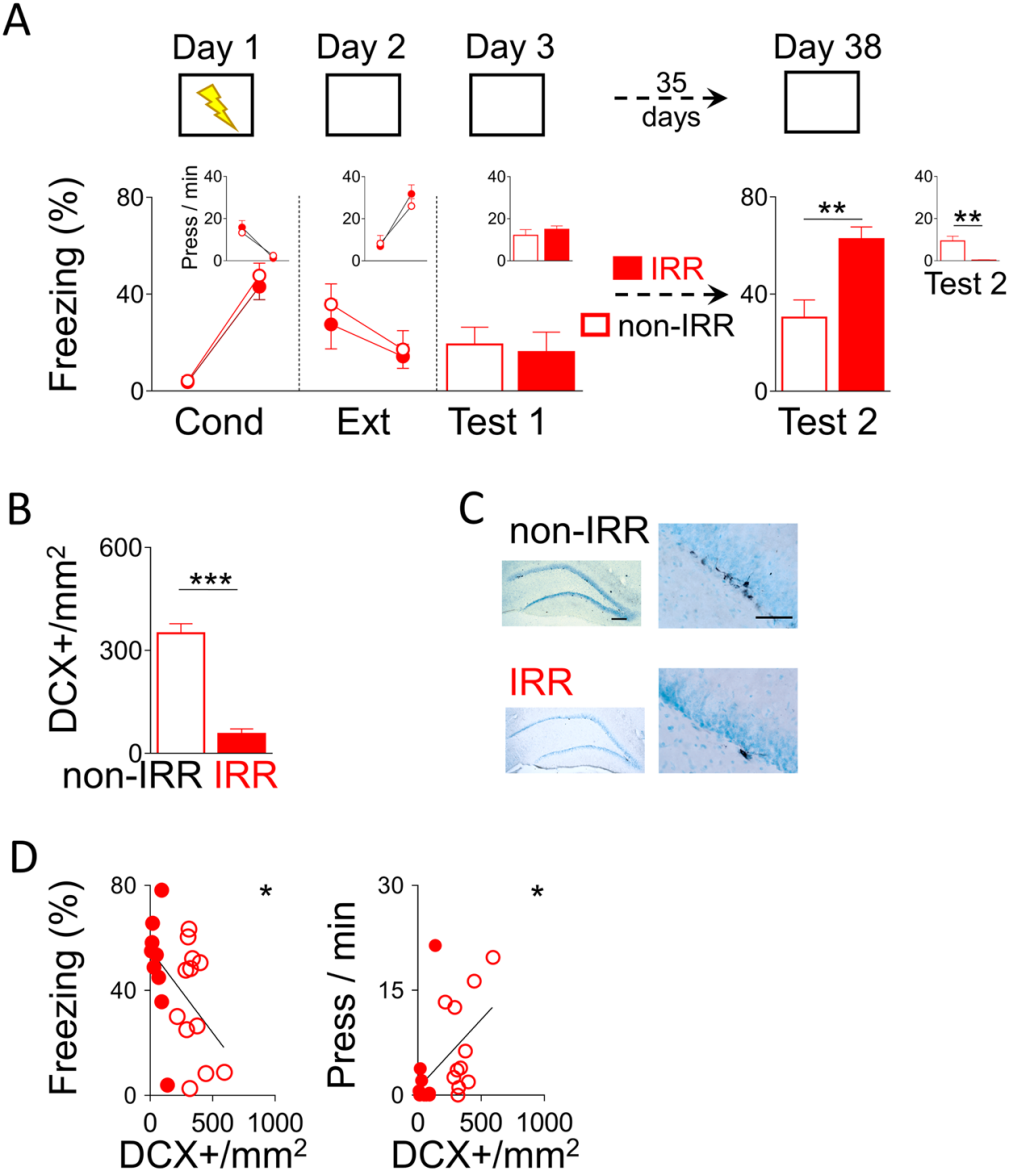
IRR-mediated neurogenesis ablation after extinction promoted spontaneous fear recovery. (**A**) Top, Rats were trained to associate a context with foot-shocks (day 1; conditioning). One day later, rats were trained to associate the same context with the absence of foot-shock (day 2; extinction). The next day, rats were placed in the same context without foot-shocks (day 3; test 1). Then, rats received three IRR or non-IRR sessions during a 35-day period. Finally, rats were placed again in the same context alone to test for fear recovery (day 38; test 2). Bottom, IRR after extinction training promoted spontaneous fear recovery, as indicated by high freezing and low bar presses per minute (inset), when comparing IRR with non-IRR (IRR: n = 9; non-IRR: n = 12). Insets: bar-pressing behavior (press/min) across the different phases of the behavioral protocol. Data are shown for first block (early) and last block (late) of conditioning and extinction (5-minute blocks), and as a single block in test sessions (10-minute block). (**B**) Rats subjected to IRR showed less number of immature cells (DCX+) per area (mm^2^) in the dentate gyrus of the hippocampus than non-IRR. (**C**) Representative immunohistochemically stained immature cells (DCX+; black) in the dentate gyrus of the hippocampus after IRR or non-IRR. Low magnification scale bar = 200 µm and high magnification scale bar = 50 µm. (**D**) Negative correlation between DCX+/mm^2^ and percent freezing response in Test 2 (R = −0.58; *p* < 0.05). Positive correlation between number of DCX+/mm^2^ and presses per min in Test 2 (R = 0.47; *p* < 0.05). **p < 0.01, ***p < 0.001. Error bars indicate SEM. IRR: irradiation; Cond: conditioning; Ext: extinction; DCX+: doublecortin positive cells.

On day 38, over a month after the first retrieval test, to evaluate spontaneous recovery of fear, rats were tested in the same context for a second retrieval test (test 2; Fig. 2A). We found that rats treated with IRR showed significantly higher levels of fear-related responses during test 2 as compared to rats treated with non-IRR (freezing: IRR: 62.64%; non-IRR: 30.34%; *t*_(19)_ = 3.41, *p* = 0.003**;** bar-pressing: IRR: 0.31 press/min; non-IRR: 9.39 press/min; *t*_(19)_ = 3.41, *p* = 0.003), suggesting promotion of fear recovery. Importantly, and consistent with previous reports^25^, irradiated rats showed a robust decrease in hippocampal neurogenesis as compared to non-IRR rats, as indicated by significantly lower DCX+ neurons in the hippocampus of IRR-treated rats compared to non-IRR control rats (Fig. 2B,C; IRR: 350 DCX+/mm^2^; non-IRR: 56.85 DCX+/mm^2^; *t*_(19)_ = 8.40, *p* < 0.0001). Further, we found that this effect was due to specific local increase of hippocampal neurogenesis as indicated by lack of effect on neurogenesis in the rostral migratory stream (non-IRR: 0.64 ± 0.81 OD, IRR: 0.56 ± 0.06 OD; *t*_(14)_ = 0.34, *p* = 0.74). Further, we found that during test 2, fear response levels and levels of neurogenesis were related, as indicated by DCX+ neurons correlation with freezing (R = −0.58, *p* = 0.03) and bar-pressing (R = 0.47, *p* = 0.03) (Fig. 2D). Together, these findings indicate that IRR-mediated ablation of hippocampal neurogenesis after extinction promotes fear recovery.

### Neurogenesis-mediated fear recovery is associated with opposite changes in brain network activity

**Figure 3:**
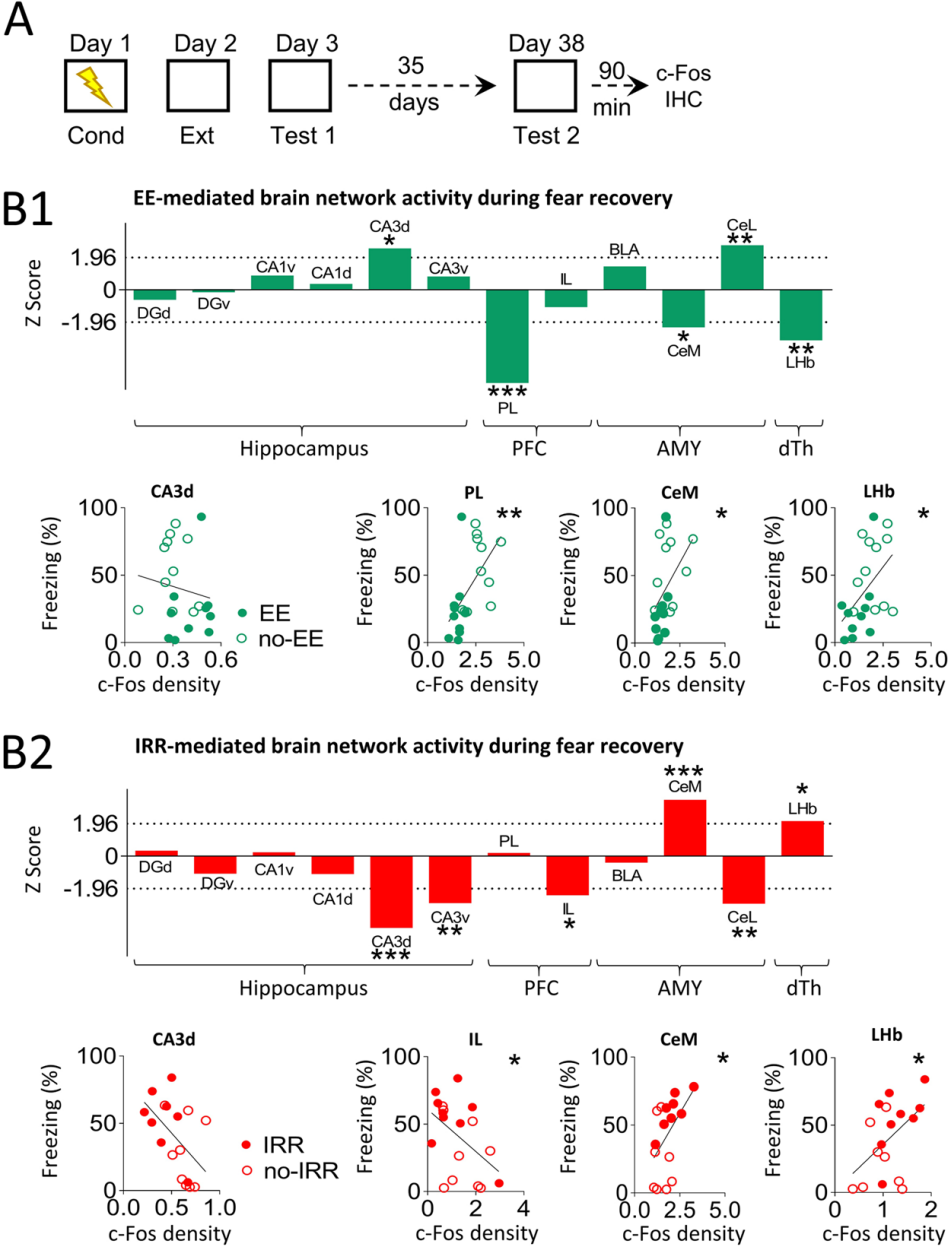
Opposing brain network recruitment during fear recovery mediated by neurogenesis manipulations. (**A**) Top, Rats were trained to associate a context with foot-shocks (day 1; conditioning). One day later, rats were trained to associate the same context with the absence of foot-shocks (day 2; extinction). The next day, rats were placed in the same context without foot-shocks (day 3; test 1). Then, separate groups of rats received different treatments to induce opposing neurogenesis effects (EE to enhance it or IRR to ablate it) during a 35-day period. After neurogenesis manipulations rats were placed again in the same context alone to test for fear recovery (day 38; test 2). Ninety minutes following this test, brains were removed and expression of activity-regulated gene *c-fos* was evaluated immunohistochemically. (**B**) Data represent normalized contrast of c-Fos immunoreactive cells in treated rats (EE and IRR) as compared to its control group (non-EE and non-IRR, respectively) (z-score). (**B1**) Top, Z-scores of c-Fos expression shows that EE, as compared to non-EE, increased recruitment (positive z-scores that exceeded 1.96; p < 0.05) of dorsal CA3 (CA3d) and centrolateral amygdala (CeL), while decreasing neuronal activity (negative z-scores that exceeded −1.96; p < 0.05) in prefrontal (PFC) prelimbic cortex (PL), centromedial amygdala (CeM) and lateral habenula (LHb). Bottom, Correlation analysis between percent freezing response during fear recovery (day 38) and c-Fos density (c-Fos positive cells per 100 µm^2^) shows a positive correlation for PL (R = 0.57; *p* < 0.01), CeM (R = 0.45; *p* < 0.05) and LHb (R = 0.48; *p* < 0.05). (**B2**) Top, Z-scores of c-Fos expression shows that IRR, as compared to non-IRR, increased recruitment (positive z-scores that exceeded 1.96; p < 0.05) of centromedial amygdala (CeM) and lateral habenula (LHb), while decreasing neuronal activity (negative z-scores that exceeded −1.96; p < 0.05) in prefrontal (PFC) infralimbic cortex (IL), dorsal and ventral CA3 (CA3d and CA3v) as well as centrolateral amygdala (CeL). Bottom, Correlation analysis between percent freezing response during fear recovery (day 38) and c-Fos density (c-Fos positive cells per 100 µm^2^) shows a positive correlation for LHb (R = 0.48; *p* < 0.05), CeM (R = 0.49; *p* < 0.05) and a negative correlation for CA3d (R = −0.51, *p* < 0.05) and IL (R = −0.47, *p* < 0.05). Both in z-score and correlation analysis: *p < 0.05, **p < 0.01, ***p < 0.001.

Our results of the manipulation of neurogenesis levels after extinction, using EE to increase it and IRR to ablate it, suggest that the amounts of adult-born neurons that are incorporated into the hippocampus is crucial to regulate spontaneous fear recovery. During fear recovery, both fear and extinction memories compete for the control of fear-related behaviour^21^. Interestingly, recent studies show that neurogenesis modifies preexisting memories^10^. One way to evaluate whether neurogenesis manipulations modify preexisting memories is by assessing the recruitment of neuronal activity in structures involved in signalling threat and extinction memories^26^. To address this recruitment issue, rats from the previous experiments (EE vs. non-EE and IRR vs. non-IRR) were sacrificed and perfused 90 minutes after the second retrieval test (day 38). We used expression of the *c-fos* immediate early gene as a marker of recent neuronal activity (Fig. 3A). To directly compare c-Fos protein expression between candidate brain structures (hippocampus, amygdala, prefrontal cortex and dorsal thalamus) and experiments (EE-mediated increase of neurogenesis and IRR-mediated ablation of neurogenesis), data were normalized using z-scores. On the one hand, we found that increased neurogenesis mediated by EE, (as compared with non-EE) was associated with c-Fos expression changes in specific brain sub-regions related to threat and extinction signalling. This is indicated by the increase of c-Fos activity in the lateral region of the central amygdala (CeL) and dorsal cornu ammonis 3 (CA3d), as well as decreased c-Fos activity in the prelimbic region of the prefrontal cortex (PL), medial region of the central amygdala (CeM) and lateral region of the habenula (LHb) (Fig. 3B1; CeL: z = 2.59, p = 0.01; Ca3d: z = 2.40, p = 0.016; PL: z = −5.52, p < 0.001; CeM: z = −2.16, p = 0.03; LHb: z = −2.94, p = 0.003). This pattern of brain activity suggests that prevention of fear recovery behaviour, mediated by increased neurogenesis, recruits an amygdala subregion associated with decreased fear-related behaviour (CeL)^27,28^, while at the same time engages inhibition of brain regions associated with increased fear-related behaviour (PL^29,30^, CeM^27^ and LHb^31^). Consistent with this interpretation, we found that brain activity pattern and freezing behaviour were inversely correlated (negative correlation) with CeL (R = −0.44, *p* = 0.047) and were directly correlated (positive correlation) with PL, CeM and LHb (PL: R = 0.57, *p* = 0.006; CeM: R = 0.45, *p* = 0.042; LHb: R = 0.48, *p* = 0.03) (Fig. 3B1).

On the other hand, we found that neurogenesis ablation mediated by IRR (as compared with non-IRR) was associated with c-Fos expression changes in brain regions related to fear and extinction memories, as indicated by increases of c-Fos activity in CeM and LHb, as well as decreased c-Fos activity CA3d, CA3v, IL and CeL (Fig. 3B2; CeM: z = 3.29, *p* = 0.0009; LHb: z = 2.01, *p* = 0.044; CA3d: z = −4.21, *p* < 0.0001, CA3v: z = −2.72, *p* = 0.006; IL: z = −2.24, *p* = 0.024; CeL: z = −2.75, *p* = 0.006). This pattern of brain activity suggests that promotion of fear recovery behaviour, mediated by ablated neurogenesis, recruits amygdala and dorsal thalamus subregions associated with increased fear-related behaviour (CeM^32^ and LHb^33^), while at the same time engages inhibition of brain regions associated with decreased fear-related behaviour (IL^34^, CeL^32^ and CA3^35^). Consistent with this interpretation, we found that brain activity pattern and freezing behaviour were related, as indicated by direct correlation (positive correlation) of freezing behaviour and c-Fos expression in CeM and LHb (CeM: R = 0.49, *p* = 0.041; LHb: R = 0.49, *p* = 0.041), as well as inverse correlation (negative correlation) in IL, CA3 and CeL (IL: R = −0.47, *p* = 0.045; CA3d: R = −0.51, *p* = 0.03; CeL: R = −0.29, *p* = 0.26) (Fig. 3B2). Together, these findings indicate that neurogenesis manipulations to increase (using EE) and ablate (using IRR) neurogenesis after extinction modify, in opposing manner, the neuronal activity of brain subregions related to preexisting fear and extinction memories.

### Neurogenesis after extinction is necessary for environmental enrichment-mediated prevention of fear recovery

**Figure 4:**
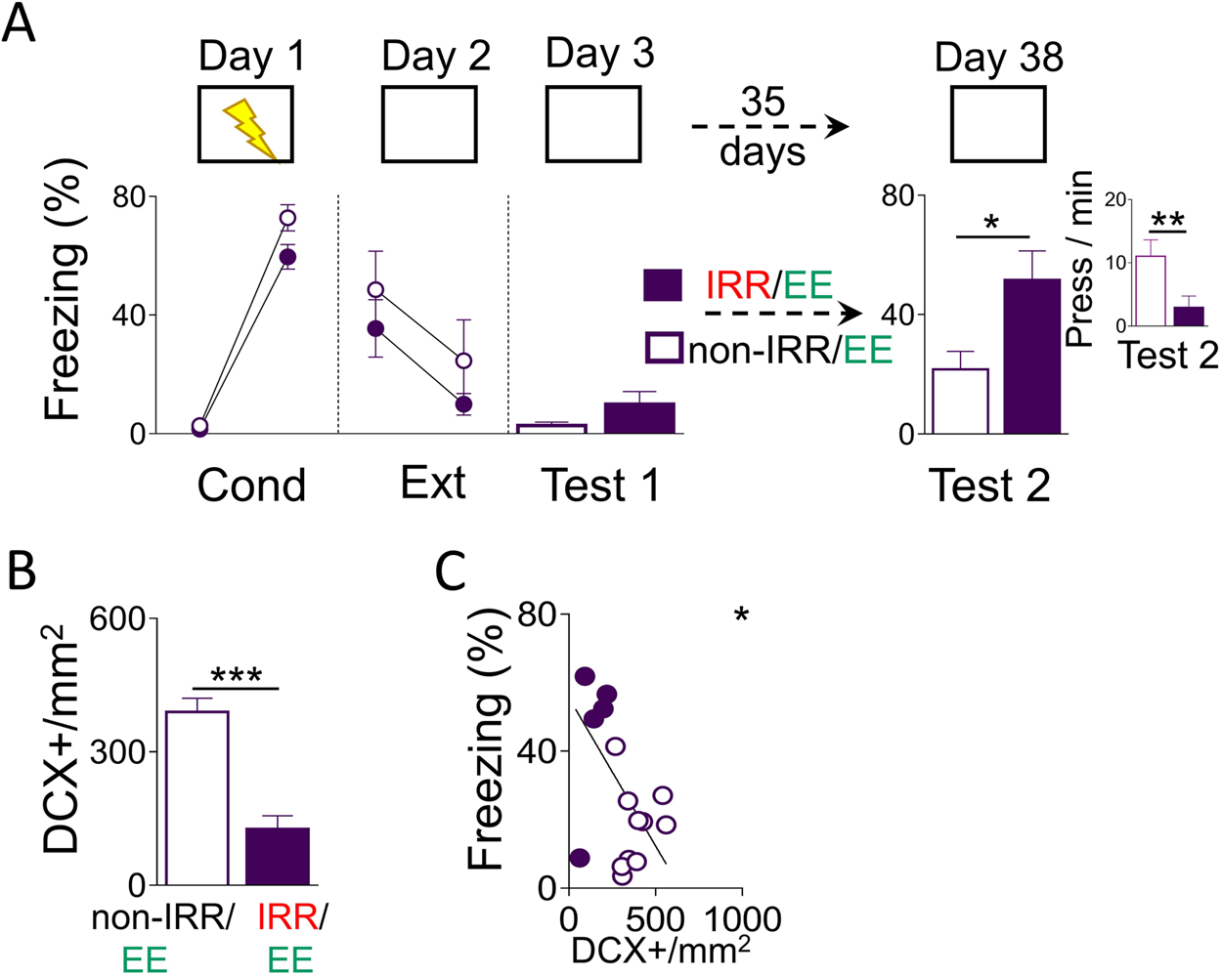
Irradiation arrest of EE-mediated neurogenesis increase after extinction restores spontaneous fear recovery. (**A**) Top, Rats were trained to associate a context with foot-shocks (day 1; conditioning). One day later, rats were trained to associate the same context with the absence of foot-shock (day 2; extinction). The next day, rats were placed in the same context without foot-shocks (day 3; test 1). Rats were housed for over a month (35 days) in an EE. At the beginning of EE rats received three IRR or non-IRR sessions. Finally, rats were placed again in the same context alone to test for fear recovery (day 38; test 2). Bottom, rats subjected to both IRR and EE (IRR/EE: n = 6) after extinction showed high spontaneous fear recovery, as indicated by high freezing and low bar pressing rate (inset) compared with rats subjected to both non-IRR and EE (non-IRR/EE: n = 12) after extinction. Data are shown for first block (early) and last block (late) of conditioning and extinction (5-minute blocks), and as a single block in test sessions (10-minute block). (**B**) Rats subjected to non-IRR/EE showed more number of DCX+ per area (mm^2^) in the hippocampus than EE/IRR-treated rats. (**C**) Negative correlation between number of number of DCX+ per area (mm^2^) and percent freezing response in Test 2 (R = −0.57; *p* < 0.05). *p < 0.05, **p < 0.01, ***p < 0.001. Error bars indicate SEM. EE: environmental enrichment; IRR: irradiation; Cond: conditioning; Ext: extinction; DCX+: doublecortin positive cells.

We showed that EE-mediated neurogenesis increase after extinction prevents spontaneous fear recovery and IRR-mediated neurogenesis ablation after extinction promotes spontaneous fear recovery. However, despite hippocampal neurogenesis being one of the most notorious effects observed at molecular level due to EE^36^, living in an EE could affect brain activity that is not limited to the hippocampus^37^. To exclude the possibility that the observed EE-mediated prevention of fear recovery is independent of hippocampal neurogenesis, we subjected rats with ablated hippocampal neurogenesis (using IRR) to an EE for over a month (35 days) after extinction and evaluated spontaneous fear recovery (day 38). As in our previous experiments in this study, rats were matched for similar levels of fear-related behaviour during the first retrieval test (day 3) (freezing: *t*_(14)_ = −1.26, *p* = 0.23; bar-pressing: *t*_(14)_ = 0.76, *p* = 0.45). This matching resulted in similar fear-related response levels between groups during conditioning (day 1; freezing: *F*_(1,14)_ = 0.47, *p* = 0.49; bar-pressing: *F*_(1,14)_ = 0.02, *p* = 0.88) and extinction (day 2; freezing: *F*_(1,14)_ = 0.32, *p* = 0.58; bar-pressing: *F*_(1,14)_ = 2.271, *p* = 0.15). We found that, rats treated with IRR followed by EE (IRR/EE) showed significantly higher levels of fear-related responses during test 2 as compared to rats treated with non-IRR followed by EE (non-IRR/EE) (Fig. 4A; freezing: IRR/EE: 51.66%; non-IRR/EE: 21.7%; *t*_(14)_ = 2.79, *p* = 0.015; bar-pressing: IRR/EE: 2.96 press/min; non-IRR-EE: 11.04 press/min; *t*_(14)_ = 220, *p* = 0.045), excluding the possibility that the observed EE-mediated effect on prevention of fear recovery is independent of hippocampal neurogenesis. Further, we confirmed that IRR decreases hippocampal neurogenesis as compared to non-IRR rats, as indicated by significantly lower DCX+ neurons in the hippocampus of IRR/EE-treated rats compared to non-IRR/EE control rats (Fig. 4B; IRR/EE: 126.34 DCX+/mm^2^; non-IRR/EE: 389.62 DCX+/mm^2^; *t*_(14)_ = 5.66, *p* < 0.0001); and that this neurogenesis ablation effect was specific to the hippocampus as indicated by lack of effect on neurogenesis in the rostral migratory stream (non-IRR: 0.58 ± 0.03 OD, IRR: 0.62 ± 0.02 OD; *t*_(10)_ = 0.77, *p* = 0.45). Consistently, we found that during test 2, freezing response and levels of neurogenesis are related, as indicated by DCX+ neurons correlation with freezing responses (freezing: R = −0.57, *p* = 0.02) (Fig. 4C). Together, these findings indicate that EE-mediated increase in neurogenesis after extinction, that in turn prevents spontaneous fear recovery, is dependent on hippocampal neurogenesis.

### Neurogenesis manipulations before extinction do not affect fear recovery

**Figure 5:**
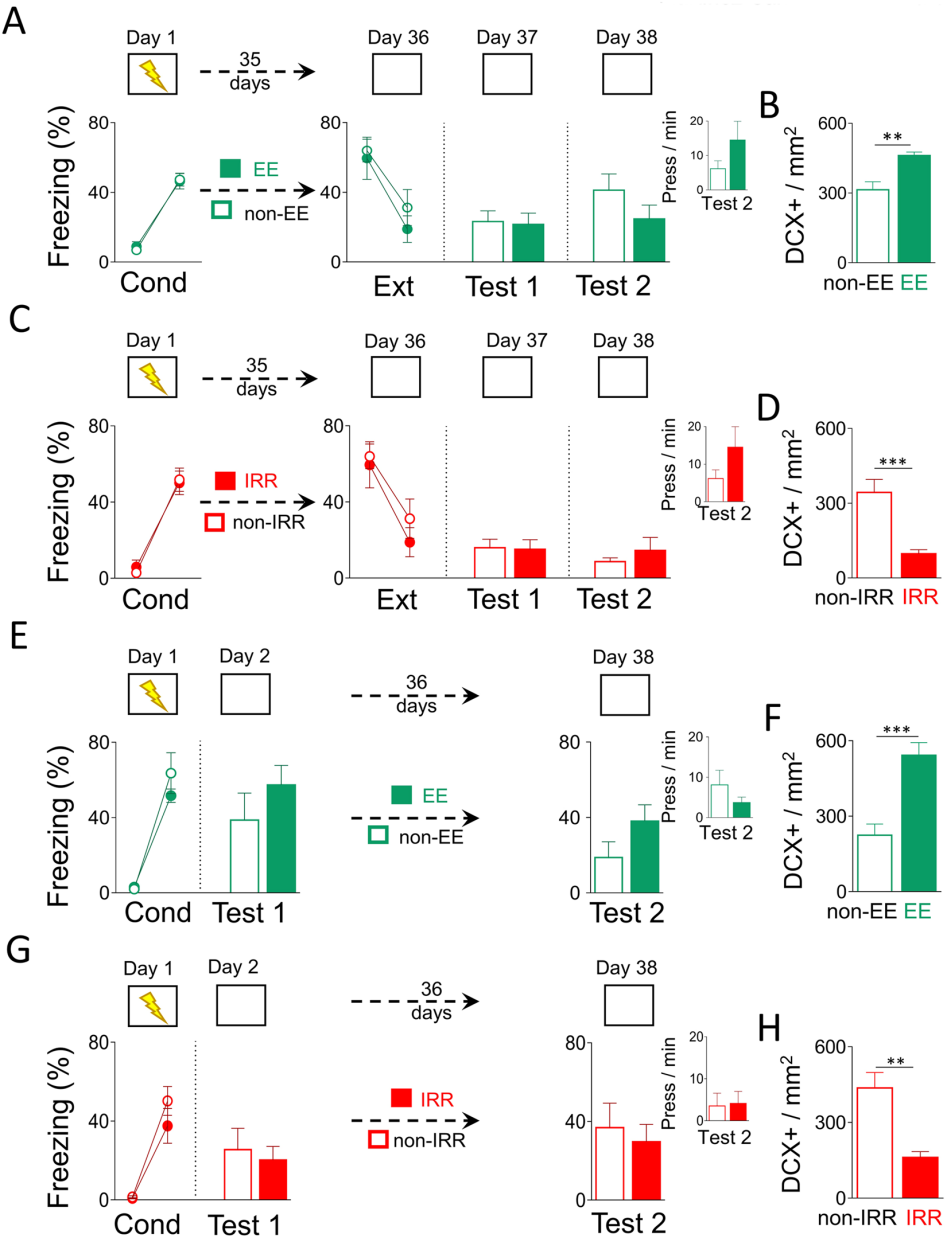
Neurogenesis increase by EE or decrease by IRR before or without extinction training does not alter fear-related behaviors. (**A**,**C**) Top, Rats that previously associated a context with foot-shocks (day 1; conditioning) were either housed in an EE or non-EE, or subjected to three IRR or non-IRR sessions, in a 35 day-period. On day 36, rats were exposed to the conditioning context without foots-shocks (extinction). The following two days, rats were exposed again to the same context alone (days 37 and 38; tests 1 and 2). Data are shown for first block (early) and last block (late) of conditioning and extinction (5-minute blocks), and as a single block in test sessions (10-minute block). (**A**) Bottom, EE before extinction training did not alter fear-related behaviors during extinction or test sessions, as compared to non-EE (EE: n = 8; non-EE: n = 8), as indicated by similar freezing responses and bar presses per minute (inset). (**B**) Rats that lived in an EE for over a month, before extinction training, showed more number of DCX+ per area (mm^2^) in the hippocampus than non-EE. (**C**) Bottom, IRR before extinction training did not alter fear-related behaviors during extinction or test sessions, as compared to non-IRR (IRR: n = 7; non-IRR: n = 6), as indicated by similar freezing responses and bar presses per minute (inset). (**D**) Rats that were subjected to IRR, before extinction training, showed less number of DCX+/mm^2^ in the hippocampus than non-IRR. (**E,G**) Rats that were trained to associate a context with foot-shocks (day 1; conditioning) were exposed to the conditioning context alone the next day (day 2; test 1). Then after over month (36 days) of either living in an EE or non-EE, or subjected to three IRR or non-IRR sessions, rats were again exposed again to the conditioning context alone (day 38; test 2). (**E**) Bottom, EE after conditioning and a retrieval test (without extinction) did not alter fear-related behaviors during test sessions, as compared to non-EE (EE: n = 8; non-EE: n = 6), as indicated by similar freezing responses and bar presses per minute (inset). (**F**) Rats that lived in an EE for over a month, after conditioning and a retrieval test (without extinction), showed more number DCX+/mm^2^ in the hippocampus than non-EE. (**G**) Bottom, IRR after conditioning and a retrieval test (without extinction) did not alter fear-related behaviors during test sessions, as compared to non-IRR (IRR: n = 6; non-IRR: n = 6), as indicated by similar freezing responses and bar presses per minute (inset). (**H**) Rats that were subjected to IRR, after conditioning and a retrieval test (without extinction), showed less number of DCX+/mm^2^ in the hippocampus than non-IRR. Data are shown for early and late conditioning and extinction (5 min blocks) and for test sessions (10 min average). **p < 0.01., ***p < 0.001. EE: environmental enrichment; IRR: irradiation; Cond: conditioning; Ext: extinction; DCX+: doublecortin positive cells.

In the present study, we have shown that neurogenesis manipulations after extinction learning, regulates the expression of spontaneous fear recovery: increased neurogenesis leads to low fear recovery and decreased neurogenesis leads to high fear recovery. However, it is possible that neurogenesis manipulations at other time points, besides after extinction, also have an impact on fear-related behaviour. To test this possibility, neurogenesis manipulations (EE-mediated increase or IRR-mediated ablation of neurogenesis) were performed before, rather than after extinction learning. Rats were matched for similar fear-related behaviour levels during conditioning (day 1; freezing: EE vs non-EE: *F*_(1,14)_ = 0.02, *p* = 0.88; IRR vs non-IRR: *F*_(1,11)_ = 0.015, *p* = 0.9; bar-pressing: EE vs non-EE: *F*_(1,14)_ = 0,32, *p* = 0.84; IRR vs non-IRR: *F*_(1,11)_ = 1.25, *p* = 0.28). The next day four different groups of rats were subjected to four different treatments: EE or non-EE and IRR or non-IRR. Over a month (35 days) after conditioning, rats extinguished fear to the context (day 36) and were tested for retrieval in two consecutive days (days 37 and 38). We found that neither increasing nor decreasing neurogenesis (using EE (Fig. 5A) or IRR (Fig. 5C), respectively) before extinction (after conditioning) affected fear expression, extinction learning or spontaneous fear recovery, as indicated by similar levels of fear-related behaviour during extinction between groups (freezing: EE vs non-EE: *F*_(1,14)_ = 0.85, *p* = 0.37; IRR vs non-IRR: *F*_(1,11)_ = 0.84, *p* = 0.38; bar-pressing: EE vs non-EE: *F*_(1,14)_ = 0.42, *p* = 0.84; IRR vs non-IRR: *F*_(1,11)_ = 0.14, *p* = 0.71) and two retrieval tests (test 1; freezing: EE vs non-EE: *t*_(14)_ = 0.18, *p* = 0.88; IRR vs non-IRR: *t*_(11)_ = 0.11, *p* = 0.91; bar-pressing: EE vs non-EE: *t*_(14)_ = 0.28, *p* = 0.77; IRR vs non-IRR: *t*_(11)_ = 0.46, *p* = 0.65; test 2: freezing: EE vs non-EE: *t*_(14)_ = 1.353, *p* = 0.11; IRR vs non-IRR: *t*_(14)_ = 1.41, *p* = 0.18; bar pressing: EE vs non-EE: *t*_(11)_ = 0.88, *p* = 0.39; bar-pressing: IRR vs non-IRR: *t*_(11)_ = 1.57, *p* = 0.14). Yet, as expected, we found significantly more DCX+ neurons in EE-treated rats as compared to non-EE rats (Fig. 5B; *t*_(14)_ = 3.88, *p* = 0.043) and significantly less DCX+ neurons in IRR-treated rats as compared to non-IRR rats (Fig. 5B; *t*_(11)_ = −4.82, *p* = 0.02), supporting the notion that EE increases neurogenesis and IRR ablates it, independently of when it occurs. Together these results indicate that for neurogenesis to influence spontaneous fear recovery it must occur after, but not before, extinction learning.

To assess whether the neurogenesis-mediated effect on fear expression we observed (low with EE and high with IRR) is dependent on the experience of extinction, rats were tested in a behavioural protocol that includes conditioning (day 1) and two different retrieval tests (day 2 and 38) separated by over a month (35 days), but without an extinction training session. After proper group matching during the first conditioning retrieval test (day 2; EE vs non-EE: *t*_(12)_ = 1.08, *p* = 0.3; IRR vs non-IRR: *t*_(10)_ = 0.4, *p* = 0.69), we found that neither EE nor IRR altered fear expression, as indicated by similar fear-related behaviours during the second retrieval test (day 38) (Fig. 5E,G; freezing: EE vs non-EE: *t*_(12)_ = −1.55, *p* = 0.15; IRR vs non-IRR: *t*_(10)_ = 0.46, *p* = 0.64; bar-pressing: EE vs non-EE: *t*_(12)_ = 0.1.26, *p* = 0.23; IRR vs non-IRR: *t*_(10)_ = 0.14, *p* = 0.88). Again, despite not having a behavioural effect, both manipulations used (EE and IRR) were effective at modifying neurogenesis levels (increase and ablate, respectively) (Fig. 5F,H; EE vs non-EE: *t*_(12)_ = 4.54, *p* = 0.007; IRR vs non-IRR: *t*_(10)_ = 4.03, *p* = 0.0024). These results indicate that neurogenesis-mediated effect on fear expression is dependent on extinction. Moreover, all together these findings highlight the importance of timing neurogenesis manipulations after extinction to influence the expression of spontaneous fear recovery.

### Increased neurogenesis after extinction does not prevent cued fear recovery

**Figure 6:**
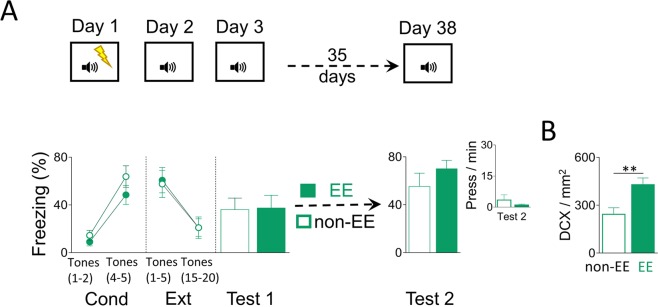
EE-mediated neurogenesis increase does not prevent tone-associated fear recovery. (**A**) Top, Rats were trained to associate a tone with foot-shock (five tone-shock pairings) (day 1; conditioning). One day later, rats were trained to associate the same tone (20 presentations) with the absence of foot-shock (day 2; extinction). The next day, rats were presented with the same tone without foot-shocks (day 3; test 1). Then, rats were housed for over a month (35 days) either in an EE or a non-EE. Finally, rats were presented with two tones alone in the same context to test for fear recovery (day 38; test 2). Data are shown for first block (early) and last block (late) of conditioning and extinction, and as a single block in test sessions (2-trial blocks). Bottom, EE after extinction training did not prevent tone-associated fear recovery, as indicated by similar freezing responses and bar presses per minute (inset), when comparing EE with non-EE (EE: n = 8; non-EE: n = 7). (**B**) Rats that lived in an EE for over a month showed more number of DCX+ per area (mm^2^) in the dentate gyrus of the hippocampus than rats that lived in a non-EE. **p < 0.01. Error bars indicate SEM. EE: enhanced environment; Cond: conditioning; Ext: extinction; DCX+ doublecortin positive cells.

Fear-related behaviour regulation can be triggered by context or specific environmental cues such as a tone. Contextual fear-related behaviour is dependent on the hippocampus, but cued fear-related behaviour is dependent on the amygdala^38^. Thus, to test whether neurogenesis-mediated prevention of spontaneous fear recovery is hippocampus dependent, we used a non-hippocampus dependent conditioning procedure (auditory fear conditioning). We compared spontaneous tone-elicited fear recovery before and after a month of either living in an enriched environment (EE) or a standard home cage (non-EE). On day 1, rats were conditioned to fear a sound (tone) by pairing it with an aversive consequence (mild foot-shocks). The next day (day 2), rats extinguished their fear to the conditioned tone by not receiving any aversive consequence. One day later (day 3), rats were tested for fear memory retrieval to the tone. After proper group matching during the first retrieval test (day 3; *t*_(13)_ = 0.91, *p* = 0.92), rats spent over a month in EE or non-EE until tested for a second retrieval test (day 38). We found that rats that lived in an EE showed similar tone-elicited fear-related behaviour levels during test 2 as compared to rats that lived in a non-EE (Fig. 6A; freezing: EE: 70.02%; non-EE: 55.36%; *t*_(13)_ = 1.15, *p* = 0.26; bar-pressing: EE: 1.031 press/min; non-EE: 3.43 press/min; *t*_(13)_ = 0.99, *p* = 0.3). Despite the consistent EE-induced increase of neurogenesis after extinction (Fig. 6B; *t*_(13)_ = 3.13, *p* = 0.008), our behavioural results indicate that neurogenesis-mediated prevention of spontaneous fear recovery is hippocampus dependent and amygdala independent.

## Discussion

We investigated when adult hippocampal neurogenesis affects spontaneous fear recovery and its underlying brain network activity. We found that EE-mediated enhancement of neurogenesis prevented fear recovery. Consistently, we found that IRR-mediated ablation of neurogenesis promoted fear recovery. Interestingly, these opposing neurogenesis manipulations affected fear-related behaviours only if manipulations occurred after, but not before or in the absence of, extinction learning. These behavioural findings indicate that neurogenesis effect on fear-related behaviour during fear recovery is time- and extinction-dependent. Notably, assessment of neural activity revealed that recruitment of distinct prefrontal-amygdala-habenula networks correlates with opposing neurogenesis manipulations and fear-related response during fear recovery. These findings add to a growing body of evidence showing that neurogenesis regulates fear-related behaviour by altering activity in fear-signalling brain regions^9,10^ and highlight the importance of timing neurogenesis manipulations after extinction learning.

New neurons are generated in the adult dentate gyrus of the hippocampus in mammals^39^; however, the functional impact of these adult-born neurons on hippocampus-dependent functions is still unclear. For instance, although the impact of neurogenesis on hippocampal-dependent contextual fear memories has been largely explored, current results are conflicting. Neurogenesis manipulations (using different methods to enhance or ablate it) can either increase^16^, decrease^10^ or have no effect^17^ on fear-related responses. Here we show that identifying *when* neurogenesis is crucial and irreplaceable may be key to understand the role it plays in fear-related behaviour. We found that neurogenesis modulations alter the of expression fear-related behaviours only if neurogenesis manipulations occurred after, but not before, extinction training, suggesting a time-dependent effect. This post-training effect is consistent with the long-held notion that the hippocampus plays a key role in memory consolidation and its retrieval^13,40^. More interestingly, our behavioural findings are consistent with the recently emerged notion that neurogenesis is crucial to alter preexisting hippocampal-dependent memories^11,41^. It has been suggested that targeting neurogenesis after memory formation (post-training) is more disruptive because these memories are already committed to memory traces^42^. Further, recent studies have shown that neurogenesis manipulations after memory formation can alter the expression of the fear memory by either promoting memory consolidation^15^ or forgetting^10^. But, we found that increasing neurogenesis after conditioning (before extinction) did not promote forgetting of fear memory. A possible explanation for the discrepancy with Akers *et al*., 2014 is twofold: stronger conditioning and saliency of bar-pressing across contexts in our study. The former explanation agrees with a recent report showing that spatial memory forgetting is prevented by long training^43^. The latter explanation agrees with another recent report showing spatial memory forgetting can be prevented by the addition of a salient retrieval reminder^44^. Although we cannot specify through which mechanism(s), our findings indicate that opposing neurogenesis manipulations timed after the formation of the extinction memory may alter the expression of fear-related contextual memories and thereby prevent or promote fear recovery.

Previous studies exploring the relationship between hippocampal neurogenesis and extinction of fear have shown conflicting data. One report found that neurogenesis ablation retards extinction learning^45^, while another found the same manipulation had no effect^46^. Yet, the effect of manipulating neurogenesis after extinction learning had not been studied. By timing neurogenesis manipulation before or after extinction and evaluating spontaneous fear recovery, our findings reveal that neurogenesis manipulations after extinction learning, but not before, is crucial to modify fear levels during fear recovery. Notably, this effect on fear-related behaviours is not present in the absence of extinction training, suggesting it is an extinction-dependent effect. Given hippocampus’ well-known role in consolidation^47^, our results extend its role to neurogenesis-mediated consolidation of extinction memory. Further, events that involve synaptic changes (including neurogenesis), mediated by hippocampal communication with the medial prefrontal cortex (mPFC) are necessary and recruited during consolidation of remote fear memory^15^. However, changes that mPFC undergoes (e.g. increased synaptic density) during remote memory consolidation^48,49^ are reverted by extinction training, suggesting synaptic modifications in mPFC can be mediated by hippocampal neurogenesis during extinction. This is consistent with the presently observed PL decreased recruitment mediated by neurogenesis enhancement and IL decreased activity mediated by neurogenesis ablation during extinction consolidation.

It is also possible that the effect we observed is also due to modulation of other cognitive functions. It has been suggested that during fear recovery of extinguished fear, two memory traces (fear memory and extinction memory) compete for the control of fear-related behaviours^1,21^. Our results suggest that increasing neurogenesis may balance towards the expression of extinction memory and decreasing neurogenesis may balance towards the expression of fear memory. Given that in our study, both fear and extinction memories share the representation of the same context, it is possible that the observed neurogenesis-mediated effect on fear recovery also relies on a neurogenesis-dependent phenomenon known as pattern separation^50^. Neurogenesis may prevent fear recovery by facilitating the separation of similar contextual representations that may otherwise cause defensive responses based on ambiguous interpretations of the valence of the context (dangerous vs. safe environment)^51^. Further experiments are necessary to test this idea formally.

But what brain activity supports the observed neurogenesis-mediated time- and extinction-dependent effect on fear recovery? Consistent with previous results, we found that extinguished fear recovery involves recruitment of the canonical brain circuit involved in fear regulation (distinct regions of the hippocampus, medial prefrontal cortex and the amygdala^26,30,34^). Additionally, consistent with habenula´s role in signalling aversion^31^, we found the habenula to be heavily correlated with fear recovery levels associated with opposing neurogenesis manipulations. Importantly, our findings suggest that modifications generated in the hippocampus mediated by adding new-born neurons modifies the activity of this circuitry. This is consistent with the idea that, through adulthood, new neurons are continuously generated and integrated into existing circuits in the hippocampus^52,53^ modulating hippocampus-dependent information processing and associated circuitry^12^. Further, our brain activity findings are consistent with studies *in silico* that predict that neuronal plasticity mediated by adding new neurons alters brain activity associated to previously established memory traces^11,54^. Thus, together our results are consistent with the idea that hippocampal neurogenesis models (or remodels) connectivity of the brain network involved in fear-related behaviours linked to previous contextual experiences.

Fear-related disorders in humans are associated with high fear relapse rates^55^, reduced hippocampal volume^56,57^, hyperactivity in the dorsal anterior cingulate cortex^58^ (a homolog of rodent PL^59^) and reduced activity in the ventromedial prefrontal cortex^60^ (a homolog of rodent IL^61^). We found that high fear recovery mediated by neurogenesis ablation after extinction is associated with decreased hippocampal and IL activity, while low fear recovery mediated by increased neurogenesis is associated with conspicuously decreased PL activity. Thus, targeting the promotion of neurogenesis (for example with regular aerobic exercise programs^62^) after extinction-based therapy may help restore activity of the fear regulation brain network and thereby prevent fear relapse in humans.

## Methods

### Subjects

A total of 107 male Wistar rats (180–220 g; Instituto de Fisiología Celular breeding colony) were housed in polyethylene cages and maintained on a standard 12 h light/dark schedule with *ad libitum* access to standard laboratory rat chow and water until bar-press training. All manipulations and behavioural procedures were performed during the light phase between 7:00 and 19:00 h. Animals were housed in groups of 2–3 per cage in a temperature-controlled environment (24 °C). All procedures were approved by the Institutional Animal Care and Use Committee of the Cellular Physiology Institute at the National Autonomous University of Mexico (FSB35–14 and FSB98-35), in compliance with the National Ministry of Health guidelines for the care of laboratory animals.

### Behaviour

Food was restricted to 18 g/day of standard laboratory rat chow until rats reached 85% of their free-feeding weight. Rats were trained to press a lever for food (dustless precision pellets, 45 mg Bioserve, New Jersey) on a variable interval reinforcement schedule (VI 60 s). All rats received six bar-press training sessions until they reached a minimum of 12 presses per minute (each session lasted 30–35 min). Pressing maintains a constant level of activity against which freezing can be reliably measure. Bar-press training, fear conditioning, extinction and retrieval tests were performed in standard operant chambers (Coulbourn Instruments, Whitehall, PA) located inside sound-isolated cubicles (Med Associates, Burlington, VT). Bar-press training and fear conditioning protocols were performed in different conditions to control for potential context pre-exposure. Bar-press and fear conditioning contexts differed considerably. While fear conditioning context included a grid floor, white house light, clear plastic walls and constant background white noise, bar-press training context included a plastic floor, no house light, striped plastic walls and no background noise. The only element in common between operant and classical conditioning contexts was the lever-feeder. Between experiments, shock grids and floor trays were cleaned with soap and water, and the chamber walls were cleaned with wet paper towels.

Contextual fear conditioning, extinction and retrieval tests were performed in the same chambers. Rats were exposed to 5 min of habituation to the context immediately followed by conditioning consisting of five foot-shocks (2 s, 1 mA; inter shock interval was variable with an average of 2 min) during 15 min exposure to the context. Contextual extinction of fear consisted of 50 min exposure to the context in the absence of foot-shocks. Contextual memory retrieval test consisted of 10 min exposure to the context alone. Food pellets were available in the conditioning chambers on a VI 60 s schedule throughout all phases of the experiment. Freezing data were calculated as mean freezing per minute and expressed in graphs as average of 5 minutes in blocks (first block (early) and last block (late) of conditioning and extinction) or a 10-minute block (test sessions). Bar-pressing data were calculated as mean bar presses per minute and expressed in graphs as 5-minute blocks (first block (early) and last block (late) of conditioning and extinction) or a 10-minute block (test sessions).

Auditory fear conditioning, extinction and retrieval tests were performed in the same chambers. Auditory conditioning consisted on the presentation of five tones (30 s, 4 kHz, 75 dB) that co-terminated with foot-shocks (1 s, 0.8 mA). Auditory extinction of fear consisted on 20 tone-presentations in the absence of foot-shocks. Auditory fear memory retrieval test consisted on the presentation of 2 tones alone. In all sessions, the interval between tones was variable with an average of 2 min. Freezing and bar-pressing during the tone were used as a measure of cued fear. Food pellets were available in the conditioning chambers on a VI 60 s schedule throughout all phases of the experiment. Freezing data were calculated as mean freezing per minute during tone presentation (trial) and expressed in graphs as average of 2 trial in blocks (first block (early) and last block (late) of conditioning and extinction, and a single block in test sessions). Bar-pressing data were calculated as mean bar presses per minute during tone presentation (trial) and expressed in graphs as 2-trial blocks (first block (early) and last block (late) of conditioning and extinction, and a single block in test sessions).

### Neurogenesis manipulations

Environmental enrichment: This procedure is used as an experimental tool to enhance adult hippocampal neurogenesis^37,62^. Rats were separated in two groups: those that lived in an enriched environment (EE) and those that lived in standard environment (non-EE). EE rats were housed in groups of 2–3 rats in standard large cages equipped with a running wheel for aerobic exercise (always available), an opaque plastic tunnel and chewing woods. Non-EE rats were also housed in groups of 2–3 rats in standard large cages but with no other material.

X–Irradiation: This procedure is used as an experimental tool to ablate adult hippocampal neurogenesis^16,25^. Rats were separated into two groups: those that were irradiated (IRR) and those that received sham-irradiation (non-IRR). All rats were anesthetized (ketamine 90 mg/kg and xylazine 10 mg/kg, i.p.). First, we obtained a cranial tomography to obtain the specific target coordinates to locally irradiate the hippocampus specifically. Then, rats were placed in a stereotaxic apparatus. The whole procedure was performed using a 6MV linear accelerator (Novalis, Brainlab-Varian). IRR rats received three arcs of circular irradiation per each side of the hippocampus; X-rays were confined to the hippocampus shape using a circular collimator (6 mm-Brainlab). Non-IRR rats were anesthetized in the same conditions and placed in the stereotaxic apparatus in the irradiation room, however, no X-irradiation was applied. For each rat, this process was repeated three times per session in three separate sessions distributed in 35 days. On each session, a dose of 5 Greys (Gy; Gy = J/kg) was applied, with a total dosage of 15 Gy for the three sessions. At the beginning and the end of each session vital signs were monitored. After each session, rats were put back in their home cages.

### Immunohistochemistry

Ninety minutes after the final behavioural test, rats were overdosed with chloral hydrate (400 mg/Kg, Sigma) and transcardially perfused with cold saline solution (0.9%), and paraformaldehyde (PFA, 4%). Brains were collected and post-fixed in the same perfusate. One week later, brains were transferred into a 30% sucrose (Sigma) in PBS. Brains were mounted and cut (50 µm) using a cryostat (Leica, CM 1520) at −25 °C. Sections were collected into tissue culture plates with antifreeze (40% glycerol, 10% ethylene glycol in PBS). Then, brain sections were mounted into gelatinized slides. On the first immunohistochemistry day, the antigen retrieval protocol was performed. Slides were placed through an alcohol gradient. Then, slides were placed in citrate buffer solution (10 mM Citric Acid, 0.05 Tween 20, pH 6.0) and subsequently in a pressure cooker during 20 min. Next, tissue underwent endogenous peroxidases blocking (Hydrogen peroxide 3%, Sigma) for 10 min. Tissue was removed from slides and placed freely floating in a blocking solution (Bovine Serum Albumin, 1%, Santa Cruz Biotechnology, NGS 1%, Jackson Immunoresearch in TBS-T) during 1 hr. Tissues were then transferred to the primary antibody (1:2500, anti c-Fos, Ab-5, Polyclonal Rabbit, Millipore/Calbiochem or 1:5000, rabbit monoclonal anti-DCX, #4604 S, Cell Signaling Technology) for 48 h at 120 rpm. The second immunohistochemistry day, sections were washed six times with TBS-T for 10 min. Then, tissue was incubated in the secondary antibody (1:1000, anti-rabbit, Jackson Inmunoresearch) for 1 h at 120 rpm. Afterwards, six TBS-T washes were performed for 10 min and then incubated in ABC complex (1:250) (kit ABC-peroxidase, elite VECTASAN®, Vector) during 1 h. Next, six more TBS-T washes for 10 min were done. DAB-Ni (Sigma) solution was used as chromogen, forming a purple-black precipitate. Sections were mounted and counterstaining with methyl green (Sigma).

### Cell counting and analysis

We quantified the number of c-Fos positive nuclei in the prelimbic (PL) and infralimbic cortex (IL), lateral habenula (LHb), basolateral (BLA) and central amygdala (CeA) as well as CA1, CA3 and dentate gyrus (DG) of the hippocampus. DCX positive cells (DCX+) were quantified in DG. The DCX+ cells were counted in all DG with a 40x objective (N.A 0.7) and images to measure the complete area were taken with 1X objective (N.A 0.04). Multiple images were captured for each brain region for c-Fos positive nuclei. All c-Fos images were taken with a microscope (Nikon Eclipse) with 10X objective (N.A 0.30) using the QCapturePro Program (7.05 v). For each region, the number of c-Fos positive nuclei were counted using the freely available image analysis software (ImageJ, 1.48 v, National Institutes of Health, US). Parameters we used to include c-Fos positive nuclei were: 52–250 µm area and 0.6–1 circularity. To directly compare c-Fos immunoreactivity in brain regions with different baselines, the expression of c-Fos was evaluated as a normalized contrast of c-Fos immunoreactive cells in treated rats (EE and IRR) relative to its control group (non-EE and non-IRR, respectively) (z-score; as in^63^). To obtain the density (numbers of cells per mm^2^) of c-Fos positive nuclei and DCX+, we obtained the weighted mean of all the cells per area unit using ImageJ.

To control for DCX staining in the X-irradiation experiments, we quantified immunostained DCX+ cells in the rostral migratory stream (RMS). RMS can be found in the region that comprises the ependymal/olfactory ventricle (E/OV) at the level of the prefrontal cortext in the rat brain. Given the high concentration and entangled configuration of immature cells in RMS, it is not possible to reliably quantify immature cells one by one using the same methods described above for the hippocampus. Thus, we used optical density (OD) values to provide a quantitative and unbiased measurement of the density of immunostained doublecortin positive (DCX+) cells in RMS. OD values represent a standardized measurement of the intensity of dark deposit in an image. To obtain an effective contrast to quantify OD, DCX+ cells were immunostained in purple/black (stains the whole cell body, dendrites and axon) whereas DCX− cells appeared as a light green nuclear counterstaining. The dramatic contrast that resulted from this staining allowed to clearly differentiate between DCX+ and DCX− congregate cells, as evidenced by very distinct grey levels in images taken with a microscope equipped with a digital camera. Finally, we converted grey levels to quantifiable standard OD unit values using a software calibration tool (ImageJ).

### Data collection and analysis

Behaviour was recorded with digital video cameras. Freezing was quantified from digitized video images using commercially available software (ANY-maze; Stoelting Co., IL, USA). The amount of time spent freezing per minute (contextual conditioning protocol) or per tone (auditory conditioning protocol) was expressed as a percentage of context exposure time or tone presentation, respectively. Bar-pressing was also used as conditioned fear-related response during context exposure or tone presentation. Groups were compared by using, when appropriate, unpaired Student’s two-tailed *t* tests or repeated-measures analysis of variance (ANOVA; STATISTICA; StatSoft, Tulsa, OK).
